# A Comparison of Social Robot to Tablet and Teacher in a New Script Learning Context

**DOI:** 10.3389/frobt.2020.00099

**Published:** 2020-10-07

**Authors:** Zhanel Zhexenova, Aida Amirova, Manshuk Abdikarimova, Kuanysh Kudaibergenov, Nurakhmet Baimakhan, Bolat Tleubayev, Thibault Asselborn, Wafa Johal, Pierre Dillenbourg, Anna CohenMiller, Anara Sandygulova

**Affiliations:** ^1^Department of Robotics and Mechatronics, School of Engineering and Digital Sciences, Nazarbayev University, Nur-Sultan, Kazakhstan; ^2^Graduate School of Education, Nazarbayev University, Nur-Sultan, Kazakhstan; ^3^CHILI Lab, École Polytechnique Fédérale de Lausanne, Lausanne, Switzerland; ^4^School of Computer Science and Engineering, University of New South Wales, Sydney, NSW, Australia

**Keywords:** human-robot interaction, child learning, language learning, social robot, cognitive learning theory, learning by teaching, interdisciplinary

## Abstract

This research occurred in a special context where Kazakhstan's recent decision to switch from Cyrillic to the Latin-based alphabet has resulted in challenges connected to teaching literacy, addressing a rare combination of research hypotheses and technical objectives about language learning. Teachers are not necessarily trained to teach the new alphabet, and this could result in a challenge for children with learning difficulties. Prior research studies in Human-Robot Interaction (HRI) have proposed the use of a robot to teach handwriting to children (Hood et al., 2015; Lemaignan et al., 2016). Drawing on the Kazakhstani case, our study takes an interdisciplinary approach by bringing together smart solutions from robotics, computer vision areas, and educational frameworks, language, and cognitive studies that will benefit diverse groups of stakeholders. In this study, a human-robot interaction application is designed to help primary school children learn both a newly-adopted script and also its handwriting system. The setup involved an experiment with 62 children between the ages of 7–9 years old, across three conditions: a robot and a tablet, a tablet only, and a teacher. Based on the paradigm—learning by teaching—the study showed that children improved their knowledge of the Latin script by interacting with a robot. Findings reported that children gained similar knowledge of a new script in all three conditions without gender effect. In addition, children's likeability ratings and positive mood change scores demonstrate significant benefits favoring the robot over a traditional teacher and tablet only approaches.

## 1. Introduction

The gradual transition of the Kazakh alphabet from Cyrillic to the Latin script was first introduced by the Kazakhstani government in late October 2017 (Altynsarina, 2018). This stage-by-stage transfer to Latin is expected to be fully implemented by 2025 (Presidential decree, 2017). Considering the explicitly formulated rationales and objectives for this reform, it is essential to pay attention to teaching all populations literacy skills in the Latin script. Even though it is thought that learning a new script will be effortless owing to the knowledge of English or other linguae mundi, there are various threats when facing the transition, such as decreased motivation to develop basic literacy skills in the Latin-based Kazakh among youth and elderly populations (Kadirova, 2018).

Since most language reforms are grounded with a certain purpose in mind, their resultant impact on literacy (Crisp, 1990), identity (Hatcher, 2008), and education in general, need to be taken into account to ease the change. Following the transfer to the Latin-based Kazakh alphabet and subsequent necessity for acquiring knowledge of the script, innovative approaches and instruments can facilitate a smooth Latin switch-over for teaching and learning. Many innovative solutions are being implemented for the purposes of educational applicability (Mubin et al., 2013) for early language and literacy learning (Neumann, 2020), handwriting learning (Hood et al., 2015), or foreign language acquisition (Balkibekov et al., 2016). For instance, Sysoev et al. (2017) presented SpeechBlocks, which is an application assisting young learners in their pursuit of mastering spelling strategies through listening to the differently positioned letters in a word. The use of this application accelerates children's engagement, self-confidence, and autonomy in learning. Furthermore, Dewi et al. (2018) developed a Javanese script learning application for Indonesian elementary age children, which made script learning easy to understand and engage with. Similarly, Yanikoglu et al. (2017) revealed that tablet-based learning supplemented by handwriting recognition and automatic evaluation was more preferred among first-graders compared to paper-based learning.

Furthermore, in recent years, research has provided a huge space for the area of language acquisition deploying social robots (Tazhigaliyeva et al., 2016; Belpaeme et al., 2018b) and this, in turn, was an impetus to the rise of human-robot interaction (HRI) as a promising research field (Mubin et al., 2013). It has created new opportunities for the integration of social robots into educational settings. To date, one of the original approaches to language acquisition and new language scripts is the Swiss-based CoWriter project. It has a clear target to assist children to learn handwriting with a social robot on the basis of learning by using a teaching approach (LbT) (Hood et al., 2015; Jacq et al., 2016; Lemaignan et al., 2016). Since the development of these studies, others have effectively employed the robot-assisted LbT approach to other fields of inquiry (Jamet et al., 2018; Yadollahi et al., 2018).

Central to our study is the CoWriting Kazakh system, which integrates a humanoid NAO robot and a tablet with a digital pen. In this scenario, the robot interacts with learners as a social partner. It is programmed to show enthusiasm to learn Kazakh language. Moreover, since the robot is programmed to speak English, the child needs to translate basic phrases from English into Kazakh (e.g., “hello—sálem”). In this way, the child takes on the role of the robot's teacher of the Kazakh language. As the children engage with the robot, they show, or “teach” the robot how to write the words in Latin-based Kazakh script. In other words, the child is recognized as a “more knowledgeable other” who leads the learning process as a teacher and peer (Vygotsky, 1980; Huong, 2007). Thus, their interaction includes a child-robot cooperation in writing words in turns where the robot's spelling of the Latin-based Kazakh words is programmed to always be correct. While not an expert, the child's expertise in comparison to the programmed robot provides an avenue for learning through teaching.

In order to investigate whether a social robot is important in the CoWriting Kazakh system, this paper aims to contribute to the literature on human-computer/robot interaction by comparing different learning aids, such as robot and tablet, tablet only, and a traditional teacher to see which teaching method is the most effective in terms of new script learning gains. We believe that by purposefully integrating an interdisciplinary lens involved in the system, inspired by pedagogy, cognitive science, and linguistics, will enhance an understanding of the research and the associated learning gains. In this sense, we have to seek out and discuss other perspectives and theories in order to offer effective learning scenarios that might increase children's learning outcomes. Thus, this paper also deals with current theories from different research fields to embed them into the CoWriter Kazakh system, evaluated by their effectiveness on children's learning experiences. This interdisciplinary nature of the study allows us to expand our understanding of a complex issue from different angles (Klein, 1990; CohenMiller and Pate, 2019). Using the human-robot interaction framework, the CoWriting Kazakh learning scenario will reduce the boundaries between various disciplinary fields and contribute to the area of new literacy studies.

## 2. Related Work

### 2.1. Transliteration and Script Learning

**Figure 1:**
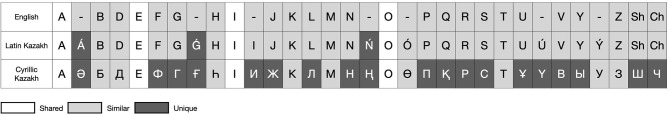
Comparison of a new Latin-based Kazakh alphabet to English and Cyrillic-based Kazakh.

In an increasingly globalized world, an English-related writing system is gaining popularity for use across languages. One language may use more than one writing system, such as the Kazakh language written both in Cyrillic and in Latin-based alphabets (see Figure 1 for a comparison between the scripts). This phenomenon is known as “digraphia,” which comprises English-related Latin (or Roman) script to constitute another language (e.g., Kazakh) (Rivlina, 2016). Roman-Cyrillic script-alternation is an example of “biscriptal” practices that are used to associate transliterated written language. For instance, the Kazakh word for naming “door” can be written either “ecik” in Cyrillic or “esik” in Latin. Rivlina (2016) broadly discussed the sociolinguistic phenomenon of employing Latin script alongside Cyrillic script to represent Russian written discourse. Building on the results of a web scraping analysis, the authors reached a conclusion that digraphic practices are used to visually draw people's attention to the written texts and to strengthen recognition and memorability by playing with words. It is also emphasized that digraphia produces translingual effects that can eliminate boundaries in terms of linguistic, national, cultural, and domain aspects.

Another study performed by Al-Azami et al. (2010) examines the effectiveness of the script conversion (i.e., transliteration) as a learning tool for writing in Bengali. In schools in London, this method is adopted to teach British-Bangladeshi students between the ages of 7–11. This Bengali-Roman biscriptal switch converts speech into text, helping children to communicate with parents and teachers, and importantly to practice a new method of increasing bilingual skills. To illustrate, if students do not recognize the correct spelling of a certain word, transliteration allows them to visualize the word, and students could grasp the meaning of the spoken word and develop their cognitive abilities. The study also showed that the use of English phonemes and converting them into a Bengali (Sylheti) script caused rapid learning. A key point in this research is that transliteration serves as a practical tool for teachers to increase students' attention span by expanding their linguistic capacities and to stimulate them to develop bilingual skills in more than one script.

Previous studies touch upon digraphia and biscriptal practices that generate social influences, however, they generally do not take into consideration an educational approach toward addressing the issues of the new script's introduction into the educational domain. So far, some methods were proposed to introduce the new alphabet to students. For instance, Gonzalez et al. (2011) experimented with two methods of tracing or copying to learn handwritten character patterns using a tablet with a stylus. It was found that two methods had differing advantages relying on short or long-term learning measures: short-term retention was better when tracing, while long-term performances had no significant difference when both methods were used. Consistent with these two methods, our study also attempts to investigate the impact of these on learning the newly-introduced Latin-based Kazakh alphabet.

### 2.2. Robot-Assisted Learning

Recent research efforts within the HRI field have shown that social robots are increasingly deployed in robot-assisted learning and education (Neumann, 2020). Robots are generally welcomed by students who view them as learning partners or companions in an optimistic way (Kennedy et al., 2016; Charisi et al., 2020). Rosenberg-Kima et al. (2019) found that the physical presence of robots brought positive changes for university students because of the technical functionality, social, and psychological activity. Namely, students pointed out the benefits as follows: “accessible to multiple people,” “immediate feedback,” “he is not judgmental like human beings,” “pleasant and motivating.” Some research has targeted specific skills required for language learning: reading (Gordon and Breazeal, 2015; Michaelis and Mutlu, 2018; Yadollahi et al., 2018), grammar (Belpaeme et al., 2018b), or vocabulary learning (Balkibekov et al., 2016). Other research demonstrated that learners cultivate favorable impressions toward robots as learning companions and the child-robot interaction may lead to increased self-confidence and better task performance requiring creativity (Dennis et al., 2016; Alves-Oliveira et al., 2017) and problem-solving (Liu and Chang, 2008). Other studies (Kanda et al., 2007; Sharkey, 2016) explored long-term learning between robots and children to better understand this type of HRI in a real-world environment.

Since 2014, the CoWriter project has investigated how robots can provide a learning environment for children in order to improve handwriting skills based on the LbT paradigm (Hood et al., 2015; Jacq et al., 2016; Lemaignan et al., 2016). This autonomous approach allows children to act as a teacher, or a tutor, who is responsible for the robot's learning. Therefore, the children, committed to the learning success of a robot, become a central actor in handwriting practices along with a social robot. In the field of pedagogy, researchers dubbed this type of process as the Protége effect in reference to Seneca's famous saying “while we teach, we learn.” In this regard, previous studies have addressed the potential benefits of LbT for learner's motivation (Jacq et al., 2016), task commitment, increased self-esteem, and mental activity (Jamet et al., 2018). In addition, Lubold et al. (2018) suggested a set of design propositions to adjust dialog strategies, revealing that individual characteristics affect the LbT outcome. Motivated by this paradigm, the CoWriting Kazakh project aims to increase children's self-confidence and motivation to learn the Latin-based Kazakh alphabet and its orthography. In view of the recent language reform in Kazakhstan, this paper investigates whether the CoWriting Kazakh project addresses challenges of teaching and motivating young learners to learn a new Latin-based Kazakh alphabet. Such findings are particularly timely as they can inform future research and practice to promote remote learning, such as required as a result of the recent COVID-19 pandemic.

### 2.3. Prior Work on CoWriting Kazakh

The CoWriting Kazakh system was previously deployed in two separate HRI studies within the novel context of learning the new Latin-based Kazakh script: an exploratory study with 48 children (Kim et al., 2019) and a follow-up study with 67 children (Sandygulova et al., 2020). Participants were asked to teach a humanoid NAO robot how to write Kazakh words using one of the scripts, Latin or Cyrillic. We hypothesized that a scenario in which the child is asked to mentally convert the word to Latin would be more effective than having the robot perform conversion itself. Two conditions were implemented that differed in who performed the conversion: Latin-to-Latin (L2L) and Cyrillic-to-Latin (C2L) conditions. In L2L, the child heard the word to be written and had to write it directly in a new Latin script. Then the robot wrote the word in Latin as corrective feedback. From this demonstration, the child is given an opportunity to see the error-free spelling in the Latin script, and importantly to learn from the robot's correct spelling via the error analysis (Jobeen et al., 2015). In C2L, the child heard the word and wrote it in a familiar Cyrillic script. Then the robot performed the script conversion by writing the same word using the Latin-based Kazakh alphabet. Results demonstrated a gender bias with the L2L strategy being more effective for girls. In contrast, boys learned significantly more when they spelled the words using Cyrillic and only observed the robot's correct spelling of the Latin-based Kazakh words. The study presented in this paper employs the L2L version of the system in order to compare what learning aid would result in greater learning gains of a new script.

### 2.4. Human Teacher vs. Robot Interaction

The shortage of teachers has become a topic for discussion across many contexts (Edwards and Cheok, 2018; Garcia and Weiss, 2019) and continues in a time where innovative technology becomes more of an imperative. Therefore, the demand for school teachers has increased exponentially and it has resulted in a necessity to recruit almost 69 million teachers to provide quality education (SDG 4) by 2030 (United Nations, 2015). This problem has led to the development of AI in education (AIEd) tools and Intelligent Tutoring Systems (ITS), which are likely to scaffold teachers in flexible and personalized ways (Luckin et al., 2016). These transformations include social robots that may be embedded into a classroom to serve the role of teacher's assistants (e.g., PaPeRo Tung, 2016, iROBI Han and Kim, 2009) by helping students to stay engaged and motivated. With ever-increasing technological advancements in education, future human teachers should focus on developing students' critical and productive thinking skills and robot assistants can minimize a teacher's workload by scaffolding the learning environment in a digitized way (Newton and Newton, 2019). This characteristic of robots is considered an asset for human teachers who may focus more on content delivery and creative instruction.

To date, robots and teachers are rarely investigated to compare their effectiveness in the classroom. Sharkey (2016) stressed that robots can act in tandem with and supplement a human teacher, but it seemed unimaginable that fully-fledged robots can be in charge of the whole learning process by themselves. Evaluating a teacher condition with and without a robot presence, Alemi et al. (2014) found children in the teacher-robot condition learned significantly more than only with a human teacher. What is worth noting here is that children can learn similarly well when the instruction is delivered either by a robot or by a teacher (van den Berghe et al., 2019). Central to the LbT approach (Lemaignan et al., 2016) employed in the present study, children tend to take on the responsibility to commit themselves for robot learning. Therefore, children's task commitment may increase in a robot condition similar to how teachers invest their time and knowledge in children's learning (Chase et al., 2009). Thus, this phenomenon is clearly important to consider as an effective approach to increase children's learning curve which needs to be supported by convincing studies in the HRI field. As of today, it seems obvious that robots can not replace teachers in classroom settings but rather act as a helpful assistant to human teachers to effectively deliver instruction. We suggest that the complementary nature of robot-assisted teaching can change ensuing dynamic technological solutions in educational settings.

### 2.5. Robots vs. Other Learning Aids

In comparison to current traditional technologies, using robots in language classrooms is stimulating, relying on their (non)verbal and social characteristics (Meghdari et al., 2013; Neumann, 2020). Unlike other computing technologies, such as tablets and laptops, the use of social robots may yield significant benefits for learning in three ways (Belpaeme et al., 2018a). First, as most learning and teaching processes happen in the classroom, robots seem a feasible option to fit the physical world and thus facilitate classroom engagement. It is highlighted that the physical embodiment of robots has a huge impact on people seeing them as more human-like, sociable, and more creative than a tablet (Li, 2015; van den Berghe et al., 2019). To illustrate, students exposed to the robot condition perceived it to be more comfortable for learning compared to the tablet condition (Rosenberg-Kima et al., 2019). Second, the presence of robots enables more social behaviors from people whose learning is not a mere task-based type of learning. For instance, Westlund et al. (2015) compared the effectiveness of three learning scenarios (human, robot, and tablet) with regard to children's rapid word learning. It was revealed that young learners strongly preferred robots despite similar word learning outcomes in three learning scenarios. No significant differences in vocabulary learning were found when robots were put on par with computers (Hyun et al., 2008). Finally, learners are more motivated and interested to learn due to the interactive communication with robots, leading to further result in an increased task commitment. Li (2015) came to the conclusion that the physical presence of a robot improves a learner's task performance compared to other learning aids.

## 3. HRI System

This section details the CoWriting Kazakh system and its scenario.

### 3.1. Software and Hardware Components

**Figure 2:**
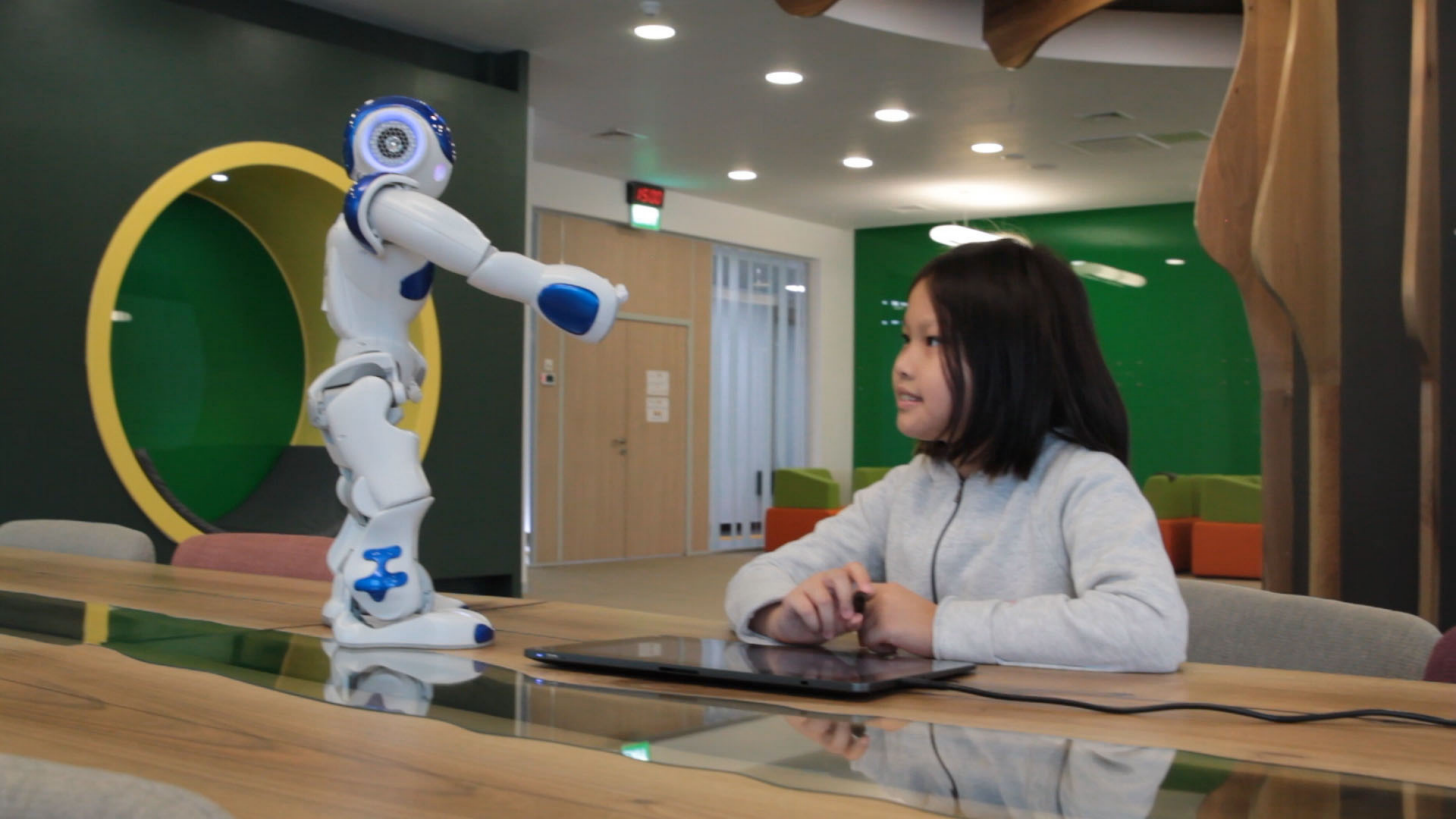
Experimental setup.

The hardware components of the system include the Wacom Cintiq Pro tablet and a humanoid robot NAO. The tablet is used as the second monitor when connected to a laptop. It is coupled with a stylus with an 8.192° of pressure sensitivity and tilting recognition. This allows us to acquire the trajectory of handwriting and the pressure and tilt at each point (Sandygulova et al., 2020). The humanoid robot NAO is an autonomous and programmable robot manufactured by SoftBank Robotics. It is the mostly used humanoid robot in HRI research for robot-assisted educational and healthcare applications. The height of the robot is 58 cm which makes it easy to transport, and its human-like appearance also attracts children. It also has 25 degrees of freedom and seven tactile sensors. In fact, CoWriting Kazakh is an extended version of the CoWriter system^1^. In comparison with the original CoWriter's LbT paradigm in which the robot's handwriting gradually improves throughout several demonstrations by the child, the CoWriting Kazakh does not include a handwriting improvement component. In the proposed system, the child-robot cooperative learning is 2-fold: (1) the robot learns new Kazakh words from the child; (2) the child learns the Latin-based Kazakh script from the robot. Their interaction takes the form of turn-taking in writing words in Kazakh (see Figure 2; Sandygulova et al., 2020).

Regarding the software aspect of the CoWriting Kazakh system, it is designed to recognize learner's handwriting in Cyrillic and transliterate it into Latin script. We developed the handwriting recognition based upon our collection of the Cyrillic-MNIST dataset (Sandygulova et al., 2020).

The interaction was implemented using NAO's English text-to-speech and face recognition engines. Across the communicative interaction, the robot demonstrated a set of animations along with hand gestures and head movements. Moreover, the robot was able to generate non-verbal social behaviors, such as recognizing the child's physical presence with eye contact. When the robot “writes,” it looks down at the tablet and moves its right hand mirroring the letters' trajectory in the air as they appear on the tablet next to child's writing. Children usually watch this “writing” motion closely, being attracted and interested by the fact that the robot can write without holding a pen or touching the surface of the tablet. The demonstration is available at the link: tiny.cc/iektpz.

### 3.2. Scenario

The scenario involves a robot taking the role of a peer. As it is introduced to a child, the peer robot is put into the position of a native English speaker who wants to learn Kazakh. Since the only alphabet known to the robot is Latin, the child is asked to show it how to write Kazakh words in the Latin-based Kazakh alphabet. The child is motivated to try their best to listen to the robot carefully in order to understand the robot's speech. It was crucial to create basic robot spoken utterances for the children's English-level appropriateness that was verified by children's English teachers.

On average, the child-robot interaction lasted 20-30 min according to how much time children take to write. The robot's list of speech utterances are as follows:

NAO: -Hello. I am a robot. My name is Mimi. [Waves his hand]

Child: -…

NAO: -I study Kazakh language. Can you help me?

Child: -…

NAO: -How do you say “Hello” in Kazakh?

Child: -Sálem

NAO: -How do you write it? Please write it using Latin letters so that I can read it.

Child: -[Writes on a tablet using Latin-based Kazakh]

NAO: -Let me try to write it too [gesticulates]. This is a correct writing using Latin letters.

… repeated for another 12 words

NAO: -You are a great teacher. Thank you very much! Goodbye! [waves].

## 4. Experiment

The methodology of the present study was developed and then aligned with the previous work (Kim et al., 2019; Sandygulova et al., 2020).

### 4.1. Method

The experiment was carried out at a primary school in Kazakhstan's capital city. It included a one-to-one interaction for each child participant. The participants were introduced to a condition in a between-subject design, with a learning aid type as the between-subject variable.

Each child interacted with a randomly selected learning aid condition for ~20–30 min. A third of the children interacted with the robot + tablet in a *Robot* condition, another third of the children interacted with a version of the CoWriting Kazakh using only a tablet in a *Tablet* condition, while the other third of the children interacted with a teacher in a *Teacher* condition using pen and paper for demonstrations. Counterbalancing was also applied in terms of gender and year group so that each condition had a balanced number of boys and girls. Assignment to each of the conditions was otherwise random for any particular child. It should be noted that when the whole experimental procedure was over (i.e., after the post-test), children from the two non-robot conditions were offered the opportunity to interact with a robot. The majority of children expressed their desire to interact with a robot. Thus, their post-test score and interview results were not affected by this interaction.

### 4.2. Recruitment

The present research project was granted approval by the Nazarbayev University Institutional Research Ethics Committee. To conduct the experiment, informed consent forms were obtained from all participants and their parents. It is supplemented by including an assent form for children and an informed consent form for their parents or legal guardians. Children were provided with an overview of the study's purpose and the data collection process. With the presence of their teachers, assent and informed consent forms were distributed to children in a classroom. Afterwards, they were asked to show the documents to their parents, and with their permission to submit them to their teachers who collected all the documents for us.

### 4.3. Participants

In total, the study recruited an equal number of 62 male and female children aged 7–9 years old. Children were assigned randomly to either a robot condition (*N* = 21), tablet condition (*N* = 21), or teacher condition (*N* = 20). The children represented different socio-economic backgrounds and all of them were native or fluent Kazakh language speakers. According to their writing experiences, second-graders had spent about 16 months writing in Cyrillic, and third-graders had spent about 28 months writing in Cyrillic before the experiment. The children learned handwriting for 6 h on a weekly basis, ranging from simple shapes to the Cyrillic alphabet after nearly 6 weeks in the first grade. In addition, they had 2 h of weekly English lessons in which they also practiced the English alphabet starting from grade one. In other words, they had spent 16 months of handwriting in English. However, the children had not been taught to write in a Latin-based Kazakh alphabet (revised version of the English alphabet with 6 distinctive letters) and its associated writing system. Therefore, compared to the Cyrillic script, all children had no learning experience in the Latin-based Kazakh alphabet.

### 4.4. Hypotheses and Conditions

Based on the CoWriting Kazakh system explained above, we examined whether it is more effective for a child to perform the mental conversion and see correctly written Latin words given by the robot. To that end, the main hypotheses are formulated as follows:

- H1: The CoWriting Kazakh will provide an effective learning scenario that will significantly improve the amount of learned letters, which, in turn, suggests that the proposed intervention contributes to learning a new script.
- H2: Girls will outperform boys in letter learning, as in our previous work we observed such gender effect in a Latin-to-Latin condition when children performed mental script conversion (Sandygulova et al., 2020).
- H3: Children will learn more letters when learning from a robot and a tablet than from a teacher and tablet only.
- H4: Children will enjoy the robot condition more in comparison to the tablet and teacher conditions, as it was reported in Li (2015), Westlund et al. (2015), and Rosenberg-Kima et al. (2019) that robots are of great advantage due to their physical presence and human-like appearance.

**Figure 3:**
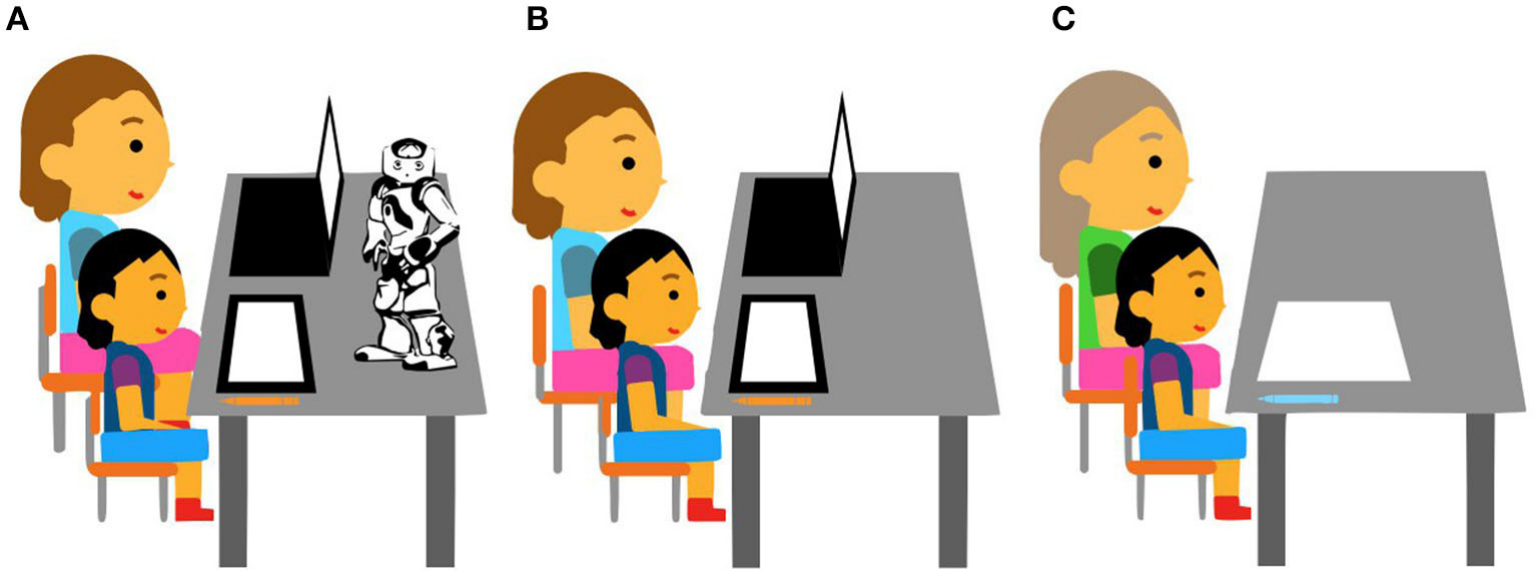
Schematic overview of the setup by conditions: **(A)** robot + tablet, **(B)** tablet only, **(C)** teacher + pen and paper.

To test these hypotheses, three conditions are distinguished with respect to the type of learning aid:

- Robot condition: the child hears the word to be written pronounced by the robot in English and has to translate it to Kazakh and write it directly in Latin on the Wacom tablet using its stylus. Then, the robot simulates the writing while the letters are written on the tablet in Latin as corrective feedback. The video demonstration is available at the link: tiny.cc/iektpz. Figure 3A presents a schematic overview of the Robot condition where a researcher controls the system launch on their computer.
- Tablet condition: the child is presented with a pop-up window on the tablet with instructions to first translate and then write the words in Latin-based Kazakh. The vocabulary is the same and 13 words are in the same order as in the Robot condition. When its time for corrective feedback, the correct spelling of the words appear in the same way on the tablet as in the Robot condition. Figure 3B presents a schematic overview of the Tablet condition where a researcher controls the launch of the system on their computer.
- Teacher condition: the teacher speaks Kazakh language and asks children to write the words in Latin-based Kazakh. The vocabulary is the same and 13 words are in the same order as in the other conditions. When it is time for corrective feedback, the teacher then shows a correctly written spelling in Latin-based Kazakh. They use a pen and paper. Figure 3C presents a schematic overview of the Teacher condition.

In all three conditions, children had to mentally perform the script conversion without help. We did not assist them in their writing process unless they did not comprehend or recognize the robot's speech. Figure 3 shows the setup of each condition.

### 4.5. Procedure

The procedure of the experiment included a survey, a pre-test, a learning activity, an interview, and a post-test. The whole process for each child took about 30–40 min.

Each child was invited from a class and accompanied by the first researcher to a place where the experiment was conducted. Before reaching the place, the first researcher began with an icebreaker to put the child at ease: “My name is Zhanel. And what is your name?” “When I was your age, I was fond of Mathematics, and what about your most liked subject?” When they entered the room, children were given a seat at the table with surveys and responded to a couple of demographic questions (i.e., age, gender) and what their mood was. Afterwards, children were sat alongside the second researcher to take a pre-test that evaluated children's existing knowledge of the Latin-based Kazakh alphabet. As the surveys and pre-tests were completed, children changed their seats and sat at the table with their learning condition (robot, tablet only, or teacher). Following the interaction, children participated in a structured interview with the first researcher who asked how they perceived their corresponding learning aid. At the end of the experiment, children were distributed a post-test analogous to the pre-test to obtain the measure of their knowledge of Latin-based Kazakh script. Similarly, the stage-by-stage procedure was followed when the first researcher accompanied the child back to the class and invited the next child.

#### 4.5.1. Survey

A mini-questionnaire was conducted by the first researcher who documented the child's demographic profile and before-the-experiment mood using a 5-point Likert scale.

#### 4.5.2. Pre-test

The pre-test was the next stage, where each child was introduced to a table of 23 Cyrillic-based Kazakh alphabet letters to complete the task by converting each letter in Cyrillic to an equivalent in the Latin-based Kazakh alphabet. This allowed us to identify the child's knowledge of Latin script before the experiment.

#### 4.5.3. Learning Activity

When the child completed the pre-test, the researcher asked the child to sit in front of the robot, tablet, or teacher. The activity would come to an end either by the child or after all 13 words were trained. As mentioned before, the words were selected in accordance with the children's level of English, which were previously approved by their English instructor. It should be noted that all 33 Latin-based letters were present in the chosen 13 words with a minimum of one letter occurrence.

#### 4.5.4. Interview

As the interaction with a robot was completed, the child took a seat along with the first researcher who then carried out a structured interview which involved the following questions from our previous studies (Kim et al., 2019; Sandygulova et al., 2020):

1. How is your mood? (5-point Likert scale)
2. Funometer scale (Markopoulos et al., 2008) was described to a child by providing an example of how it operated: the winter has the coldest weather (at the lowest level of the meter) while the summer is the sunniest season (at the highest level of the meter). How would you rate today's weather? Afterwards, the following example showed an enjoyable measurement: “imagine that you are having a birthday party and you receive many gifts, you enjoy your time very much (rate your mood at the top of the meter), or in reverse when you feel bored with waiting for a bus (rate your mood at the bottom of the meter). Similarly, how would you rate your learning activity?” (The rating was scaled from 0 to 100).
3. Sorting task: The researcher illustrated this task to a child by demonstrating five items they considered the most and least interesting. In an activity, five small paper items were presented: a book, a tablet, a NAO robot, a computer, and a teacher. (The sorted position of the child's learning aid was recorded using a 5-point Likert scale).
4. Likewise, the researcher asked the children to sort the five items with regard to what/who is the least/most effective for learning? (The sorted position of the child's learning aid was recorded using a 5-point Likert scale).
5. Children also performed a sorting task with the five items (a book, a tablet, a robot, a computer, and a teacher) responding to the question what/whom they preferred the least/most?
6. In closing, children sorted the five items based on what/who is the easiest way to learn with/from?

These questions helped to reveal how children feel about the interactions. We applied different techniques to explain the procedures explicitly and ask easy to follow questions. For instance, the Funometer scale and a paper version of the learning aids were printed for children to manually move the paper and situate it on a scale. This was an appropriate option compared to pictorial five-level Likert items by providing more detailed responses. Most children placed their ratings on a Funometer scale near 70–90 out of 100. In addition, the children were asked to rate their mood after the interaction, to compare whether their mood changed or not. Finally, we performed a group of sorting tasks in which children were asked to sort first their corresponding learning aid (e.g., teacher) and then to sort a robot as well.

#### 4.5.5. Post-test

The post-test was the final stage in the experiment. At this stage, children were introduced to the same table of 23 Cyrillic letters as distributed in the pre-test. Similarly, children were asked to write Latin-based Kazakh letters. This stage was important to evaluate the children's learning gains by comparing the number of learned letters in pre- and post-tests. Children were given a book for participation after the completion of the post-test. Children that did not get to interact with the robot were offered the opportunity to repeat the learning activity but with the robot this time. Their performance in the tests and responses to interview questions were not affected by this activity with the robot.

## 5. Results

A series of Kolmogorov-Smirnov and Shapiro-Wilk tests were conducted on all dependent variables overall and within groups (i.e., gender and condition) to check the assumption of normality. Since some scores were significantly non-normal, non-parametric tests were used for the statistical data analysis presented in some of the following sections.

### 5.1. Learned Letters

Four children did not complete their post-tests, thus this analysis was conducted on data from 58 children (see Table 1 for demographics of participants for every condition). The number of learned letters was calculated to identify the difference between letters known in the post-test and the pre-test (e.g., if 18 correct letters were marked in the post-test and 10 correct letters were marked in the pre-test, the number of learned letters is 8). As a result of the learning activity, children improved their knowledge of the Latin-based Kazakh alphabet. The average number of learned letters was 3.67 (SD = 2.37, Max = 9, Min = 0).

**Table 1:** Pre- and post-test descriptives.

**Gender**	**Condition**	**Age**	***N***	**Pre-test**	**Post-test**	**Learned letters**
Boys	Robot	8.30	8	*M* = 11.63, *SD* = 4.75	*M* = 15.50, *SD* = 5.24	*M* = 3.88, *SD* = 2.48
	Tablet	8.40	10	*M* = 12.00, *SD* = 5.06	*M* = 15.20, *SD* = 4.16	*M* = 3.20, *SD* = 3.12
	Teacher	8.36	11	*M* = 10.55, *SD* = 4.41	*M* = 14.82, *SD* = 5.17	*M* = 5.18, *SD* = 2.27
	Overall	8.36	29	*M* = 11.34, *SD* = 4.61	*M* = 15.14, *SD* = 4.69	*M* = 4.14, *SD* = 2.69
Girls	Robot	8.64	11	*M* = 11.64, *SD* = 5.59	*M* = 15.00, *SD* = 4.92	*M* = 3.36, *SD* = 1.43
	Tablet	8.36	10	*M* = 12.10, *SD* = 4.46	*M* = 14.70, *SD* = 3.59	*M* = 2.60, *SD* = 2.37
	Teacher	8.67	8	*M* = 10.63, *SD* = 4.93	*M* = 14.38, *SD* = 5.55	*M* = 3.75, *SD* = 1.91
	Overall	8.55	29	*M* = 11.51, *SD* = 4.90	*M* = 14.72, *SD* = 4.53	*M* = 3.21, *SD* = 1.92

To test H1, we conducted a paired samples t-test on pre- and post-tests which revealed that children had a statistically significant improvement in their Latin alphabet knowledge from 11.48 ± 4.64 to 14.68 ± 4.62: *t*_(57)_ = −10.5, *p* < 0.0005. Table 1 presents pre- and post-test descriptives.

**Figure 4:**
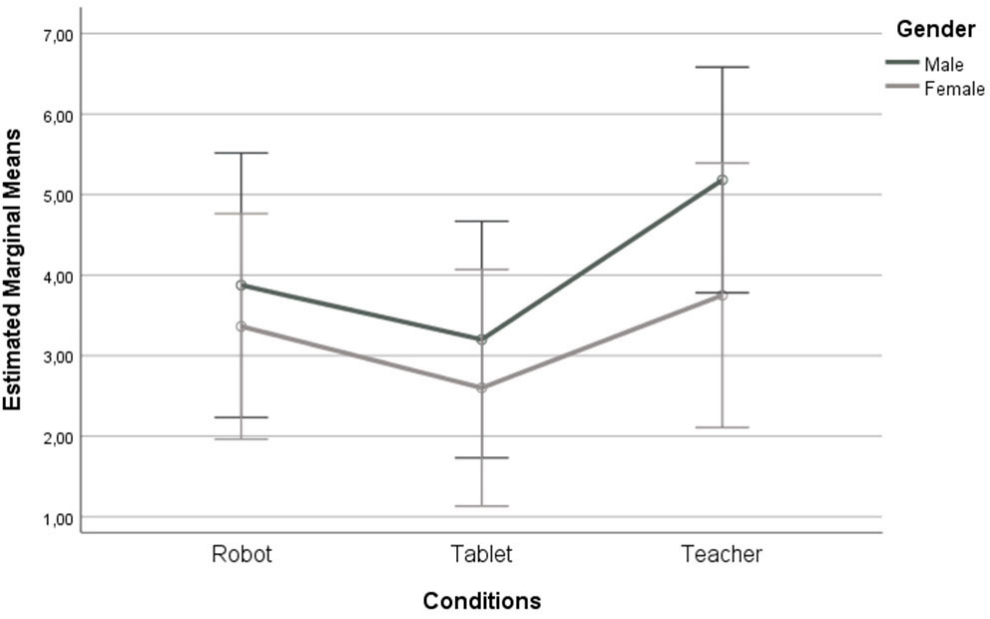
Average number of learned letters for boys and girls by the conditions. Error bars show 95% Confidence Interval.

A two-way ANOVA was conducted examining the effect of gender and condition on a number of learned letters. We did not find a statistically significant interaction between the effects of gender and condition, *F*_(2, 52)_ = 0.225, *p* = 0.799. Boys and girls learned the most letters in the Teacher condition: boys learned 5.18 ± 2.27 while girls learned 3.75 ± 1.91. The robot condition was the second most effective learning aid where boys learned 3.88 ± 2.48 and girls learned 3.36 ± 1.43 letters. The tablet condition was the least effective for both gender groups (3.2 ± 3.11 vs. 2.6 ± 2.36), though not significant. These results are presented in Figure 4.

To test H2, a Welch's ANOVA was conducted to examine whether there is a significant gender difference in the number of learned letters: *F*_(1, 50.54)_ = 2.299, *p* = 0.136. Boys learned 4.14 ± 2.69 while girls learned 3.21 ± 1.92 letters. Girls scored slightly better in a pre-test (11.51 ± 4.90 vs. 11.34 ± 4.61), but in a post-test boys outperformed girls (15.14 ± 4.7 vs. 14.72 ± 4.53), though not significantly. Then, a series of separate one-way ANOVAs was conducted to find gender differences for each condition: Teacher, Robot, and Tablet. However, the differences in learning gains were not significant between boys and girls when analyzed separately either. This finding rejects our H2, suggesting that boys and girls learned more-or-less equally, which contradicts our previous finding that the Latin-to-Latin approach was more effective for girls who learned more, as it was previously found in Sandygulova et al. (2020).

Finally, to test H3, we examined whether there is a significant difference in the number of learned letters between the three conditions. The assumption of normality was met by all three groups. Levene's test revealed that population variances of learned letters for the three types of conditions are equal, *F*_(2, 55)_ = 1.3, *p* = 0.28. As all the assumptions were met, we proceeded with a one-way ANOVA which revealed that there is no statistically significant difference in the number of learned letters between conditions: *F*_(2, 55)_ = 2.618, *p* = 0.082. Children learned slightly more letters in the Teacher condition (4.58 ± 2.19), followed by the Robot (3.58 ± 1.89), and Tablet conditions (2.9 ± 2.71), though without significance. This finding rejects our H3, suggesting that Robot, Tablet, and Teacher conditions did not lead to significantly different learning gains.

In addition, in order to find out if the three conditions were equally as effective as a learning aid, we performed an equivalence analysis TOST (two one-sided tests) test (Rusticus and Lovato, 2011; Lakens et al., 2018) setting equivalence bounds Δ_*L*_ and Δ_*U*_ to SESOI which is equal to ± *d*_*crital*_. The critical effect size was calculated using the following formula, $d_{crital}=t_{critical}\sqrt{\frac{1}{n_{1}}+\frac{1}{n_{2}}}$, that was proposed by Lakens et al. (2018). As a result, it did not show a significant equivalence: Robot vs. Tablet: *d* = 0.30, 95% CI for Cohen's d: [−0.34, 0.94], Δ_*L*_ = −0.33, Δ_*U*_ = 0.33, *t*_(37)_ = 0.35, *p* = 0.636, 90% CI for mean difference [−0.26, 1.62]; Tablet vs. Teacher: *d* = 0.713, 95% CI for Cohen's d: [−0.71, 2.35], Δ_*L*_ = −0.418, Δ_*U*_ = 0.418, *t*_(37)_ = −1.599, *p* = 0.94, 90% CI for mean difference [−1.36, −0.06]; Robot vs. Teacher: *d* = −0.535, 95% CI for Cohen's d: [−1.18, 0.11], Δ_*L*_ = −0.423, Δ_*U*_ = 0.423, *t*_(36)_ = −0.867, *p* = 0.804, 90% CI for mean difference [−1.79, −0.21]. These findings suggest that the three conditions were neither significantly different nor significantly equivalent in their facilitation of learning gains. This result is due to our sample size being quite small, leading to not having sufficient power to reject either null hypothesis.

### 5.2. Mood Change

The mood change variable was calculated as the difference between reported pre- and post-interaction ratings of children's mood on a 5-point Likert scale.

A series of Mann-Whitney U tests was conducted that revealed that there is a statistically significant difference in Mood Change score between the Robot (0.45 ± 0.68) and Teacher (−0.05 ± 0.52) and Tablet (−0.05 ± 0.83) conditions: *U* = 122, *W* = 312, *Z* = −2.35, *p* = 0.019. This finding supports our H4, in that the Robot condition was more enjoyed in comparison to the other two conditions.

A Mann-Whitney U test was conducted to check gender differences in children's Mood Change values, however it was not significant: *U* = 370.5, *W* = 805.5, *Z* = −1.184, *p* = 0.236.

Apart from the numerical value of the Mood Change variable, we also categorized it as either Increased, Decreased, or Unchanged. A series of chi-square tests of independence was conducted to examine the effect of categorical variables (gender or condition) on children's Mood Change. We did not find any statistically significant results between boys and girls for these measurements.

**Figure 5:**
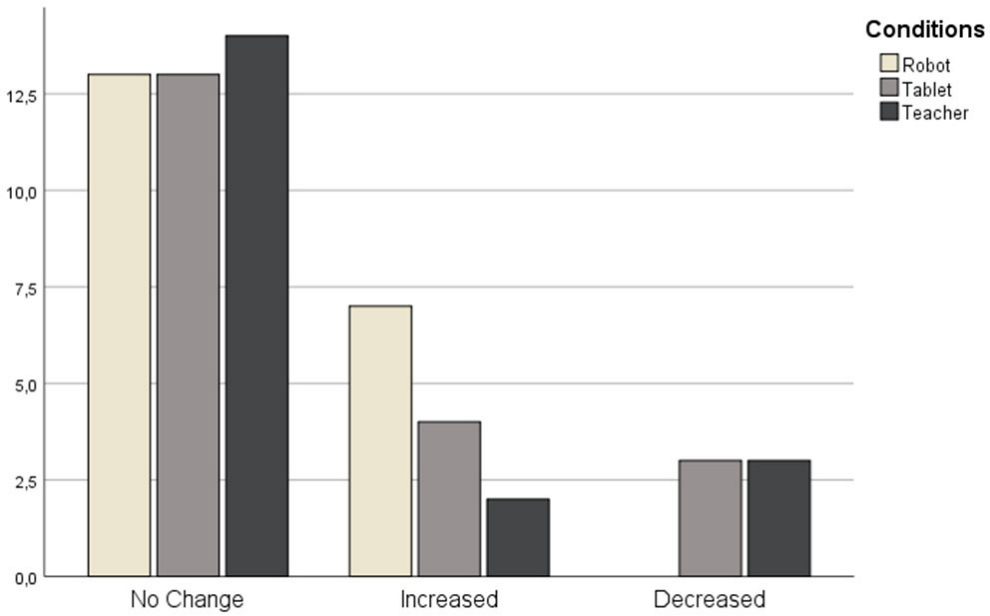
Number of children from three conditions grouped by their mood change.

There were no significant differences between conditions in how children responded to Mood Change: χ^2^ (4, *N* = 59) = 5.932, *p* = 0.204. Figure 5 presents that although there is a similar number of children who did not have their mood changed in all conditions, children in the Robot condition were more likely (7) to have their mood increased in comparison to Tablet (4) and Teacher (2) conditions. And none of the children in Robot condition had their mood decreased in contrast to three children in Tablet and Teacher conditions each.

### 5.3. Funometer

Children were asked to rate how much they enjoyed their corresponding learning activity ranging from 0 to 100 on a Funometer scale (Markopoulos et al., 2008). An average rating for all children was 78.22 ± 17.61 (*Mdn* = 75, *Max* = 100, *Min* = 40).

A Kruskal-Wallis test revealed that there is no significant difference in children's ratings between three conditions: $\chi_{\left(2\right)}^{2}=0.849,p=0.654$. Children in the Robot condition rated their experience as slightly higher (*M* = 80.75, *SD* = 17.71, *Mdn* = 77.5, *Max* = 100, *Min* = 50) than those children in the Tablet (*M* = 77.5, *SD* = 18.17, *Mdn* = 75, *Max* = 100, *Min* = 40) and Teacher conditions (*M* = 76.32, *SD* = 17.55, *Mdn* = 75, *Max* = 100, *Min* = 45), though not significantly.

We conducted a Mann-Whitney U Test to compare children's ratings between gender groups showing that boys rated their interaction as slightly better than girls did, even though not significantly: *U* = 319.5, *W* = 754.5, *Z* = −1.784, *p* = 0.074. Boys' rating was 82.17 ± 15.69 (*Mdn* = 80, *Max* = 100, *Min* = 50) while girls rated their experience as 74.14 ± 18.81 (*Mdn* = 75, *Max* = 100, *Min* = 40).

### 5.4. Sorting of Learning Aids

When asked to position children's corresponding learning aid according to its effectiveness to teach, easiness to learn from, being interesting and enjoyable in comparison with a robot, book, tablet, computer, and a teacher, children's ratings were recorded and analyzed against each other.

#### 5.4.1. Effectiveness Rate

A Kruskal-Wallis test revealed that there was a significant difference in this rating between conditions: $\chi_{\left(2\right)}^{2}=7.36,p=0.025$. A series of Mann-Whitney U tests was conducted to check between Teacher and Robot ratings: *U* = 88, *W* = 298, *Z* = −2.761, *p* = 0.006. The Teacher was rated as 3.9 ± 1.4 which was significantly higher than the Robot's rating (2.9 ± 1.1). Tablet was rated as 3.05 ± 1.47 which did not have significant difference with the Robot rating, but was significantly different with the Teacher rating: *U* = 88, *W* = 298, *Z* = −2.761, *p* = 0.006. No gender differences were found for this rating.

We also noted the sorting position of the robot for all children. We found that children who interacted with the tablet rated the effectiveness of the robot as 2.76 ± 1.00, while children who were taught by the teacher rated the robot as 2.79 ± 0.86. Participants in a robot condition rated it slightly higher at 2.9 ± 1.1. A series of Mann-Whitney *U* tests did not find a statistically significant difference in this rating, neither between conditions nor between gender groups.

#### 5.4.2. Easiness Rate

No statistically significant differences were found for this rating between different learning aids. Girls rated the learning activity significantly easier (3.82 ± 1.28) than boys did (3 ± 1.39) according to a Mann-Whitney *U* test: *U* = 285.5, *W* = 781.5, *Z* = −2.313, *p* = 0.021.

Participants from the tablet condition rated the robot as slightly more difficult (2.81 ± 1.07) than those participants that interacted with a teacher (2.79 ± 1.36) and robot conditions (3.3 ± 1.42), though not significantly. Gender groups did not rate the robot as significantly different for this rating.

#### 5.4.3. Likeability Rate

A Kruskal-Wallis test was conducted to see which learning aid was rated the most likable and showed that there was a statistically significant difference in this rating: $\chi_{\left(2\right)}^{2}=12,p=0.002$. A series of Mann-Whitney tests showed that the robot was rated as statistically significantly higher (4.35 ± 1.04) than both the tablet (3.57 ± 1.29): *U* = 127.5, *W* = 358.5, *Z* = −2.284, *p* = 0.022, and the teacher (3.06 ± 1.21): *U* = 70.5, *W* = 241.5, *Z* = −3.329, *p* = 0.001. No gender differences were found for this rating.

A series of Mann-Whitney *U* tests showed that those who interacted with the robot liked it significantly more and rated the robot as 4.35 ± 1.04 than those who interacted with the tablet only (3.57 ± 1.36): *U* = 137.5, *W* = 368.5, *Z* = −2.013, *p* = 0.044. Children from the teacher condition rated the robot as 4.11 ± 1.29, though it was not significant.

#### 5.4.4. Interest Rate

A series of Mann-Whitney U tests revealed that children rated the Robot as significantly more interesting than the Teacher: *U* = 114.5, *W* = 285.5, *Z* = −2, *p* = 0.045. The robot was rated as 3.9 ± 1.12, while the teacher was rated as 3.17 ± 0.99. No statistically significant differences were found between other learning aids as well as between gender groups.

A series of Mann-Whitney U tests revealed that children in the robot condition rated it as significantly more interesting (3.9 ± 1.12) than those in the tablet condition (3.09 ± 1.22): *U* = 135.5, *W* = 366.5, *Z* = −2.042, *p* = 0.041. No statistically significant differences were found between participants' ratings in Teacher and Robot conditions, as well as between gender groups.

## 6. Discussion and Limitations

Since all participants attended the same school, we cannot generalize our results or confidently state that the findings will be workable for other Kazakhstani schools.

However, as found in the analysis of the results, we can claim that H1 is valid, supporting that the intervention with the system was effective on the children's performance in both pre- and post-tests at a high statistically significant level (*p* < 0.001). We found similar findings in our previous studies which allow us to declare the effectiveness of the proposed learning approach of teaching in a single session. The children were able to learn from the approach when they first attempt to convert the words to Latin themselves and then observe the corrective feedback.

### 6.1. Gender Differences

Given the non-significant differences between gender groups in the presented study, we can interpret that boys and girls learned more-or-less similarly in all conditions. It contradicts our H2 and previous study's results (Sandygulova et al., 2020) in which we found a gender imbalance in the performance of boys and girls with respect to the learning gain results. Girls performed better in the Latin-to-Latin condition and learned significantly more letters. As distinct from it, boys learned more letters when following the Cyrillic-to-Latin condition. Since this study only offered the Latin-to-Latin condition, this mismatch is an unexpected turn but might be due to the different set of words that was selected for this study. This time, most of the words that the child had to show to the robot had a maximum of four letters in contrast to Sandygulova et al. (2020)'s selected words. This should be carefully accounted for in our future studies.

### 6.2. Robot vs. Human Teacher

The study revealed neither a statistically significant difference nor statistically significant equivalence in the number of learned letters when taught by a robot or by a teacher. This result is due to our sample size being quite small leading to an insufficient power to reject either null hypothesis. This resonates with the previous works that found no significant differences in the number of learned words (Westlund et al., 2015), and test-scores in mathematics with either a robot or human teacher (Mubin et al., 2019). In the meantime, significant benefits of peer robots over traditional teacher-to-student interactions and advantages of robot-assisted classes in contrast to only a teacher-led classes have been discussed so far (Alemi et al., 2014; Belpaeme et al., 2018a). In a similar vein, Rosenberg-Kima et al. (2019) also indicated that robots successfully assisted the learning experience of students, and in some cases even more effective interactions were reported in comparison with human teachers. Importantly, they also stressed the idea of Human-Robot-Collaboration (HRC) that provides a space for a human teacher and a social robot to work in tandem. Robots do have essential skills to act in the capacities of tutors and teacher's assistants, bearing in mind that human teachers cannot be fully replaced in a classroom. Considering that the comparison of the effectiveness of a human and a robot intervention is rarely explored, this study needs further refinement in a larger sample size and with longer interactions.

### 6.3. Robot + Tablet vs. Tablet Only

Similarly, the results from these two conditions fail to reject the standard null hypothesis, while failing to reject the equivalence null hypothesis, which leads us to conclude both “not different” and “not equivalent.” The non-significant difference between the two conditions can be explained by the fact that letter learning is a simple task and any exposure to this task leads to learning gains. In addition, since our learning scenario does not rely on the main advantage of social robots over the tablet, i.e., their ability to provide verbal and non-verbal cues, this might have caused the tablet only version to provide more-or-less similar alphabet learning gains. Thus, it can be noted that touch-screen tablets are a relevant option for learning a new script in line with robots. These results are reminiscent of the large-scale study (Vogt et al., 2019) which indicates that the success of learning L2 words cannot be accomplished merely with the robot condition. As a result of these findings, we can deduce that robots combined with and assisted by tablets are considered preferable rather than just the robot or tablet. For instance, instead of using them separately, Park and Howard (2013) proposed the HRI toolkit that enables the use of tablets as mediators between humans and robots. By comparing them, however, an increasing number of studies (Li, 2015; Westlund et al., 2015; Rosenberg-Kima et al., 2019) have reported that robots are of a great advantage due to their physical presence and human-like appearance compared to portable tablets. These socially-situated features of robots seem essential to the learning process compared to the passive and virtual interaction with tablets. Future work should examine the effectiveness of a robot only, a tablet, and tablet and robot conditions on the children's learning outcomes.

### 6.4. Children's Perception

Interestingly, children's self-reported ratings of their mood were different for Robot and Teacher conditions, where children's mood was increased on average by 0.45 on a 5-point Likert scale after the Robot condition, while it was decreased on average by 0.05 points after the Teacher condition. On the other hand, children rated the teacher as more effective for learning in comparison to both the robot and the tablet aids.

Aligned with the Mood Change findings, children in the Robot condition rated their Likeability sorting of their learning aid type much higher than those who interacted in the Tablet and Teacher conditions. In addition, the robot's rating for being interesting was higher than this rating for the teacher. These results favoring the robot are important, since one of our main goals is to motivate and encourage children to learn the new script. Research has shown that affective responses, such as emotion and mood, are interwoven with learning and cognition, and it is hypothesized that positive mood leads to pleasant and open-minded cognitive experiences framed within “mood-dependent cognitive styles” (Hascher, 2010). Prior work (Bryan and Bryan, 1991; Bryan et al., 1996) has shown that children in a positive mood condition performed significantly better than children in a control group. Thus, we can assume that positive mood as an affective reaction might create a favorable learning environment, resulting in the enhancement of divergent thinking and task engagement (Pekrun, 1992; Efklides and Chryssoula, 2005). In HRI, researchers have started to investigate how social robot could benefit in making learning more efficient and more enjoyable (Movellan et al., 2009; Tozadore et al., 2017; Sandygulova and O'Hare, 2018; van den Berghe et al., 2019; Chen et al., 2020). (Johal, 2020) found that more than half of the recent studies in social robots for education evaluate the affective outcomes of the robot-learner interaction; and about 30% report both cognitive and affective outcomes. More generally, humanoid robots are suggested to provide positive peer-like interaction with children, broadly promoting enjoyment through the interaction. Our study shows that children's likeability and positive mood change bring significant benefits compared to other teaching approaches. However, the relationship between enjoyment and learning outcomes is still not clear (i.e. a causality, a correlation or a more complex relationship) (Girard et al., 2013). As such, investigations are needed to assess the added value provided by robot-assisted learning (which other teaching approaches otherwise lack) as well as a follow-up longitudinal study allowing to evaluate retention outcomes. In such future research, the effect of mood should also be integrated as related to students' learning outcomes.

### 6.5. Task Difficulty

Indeed, this experiment has brought up some questions of identifying effective learning scenarios and tools for learning a new script. Future studies can focus on vocabulary choice as it might benefit children to use their foreign language vocabulary resources to improve foreign script learning (e.g., Latin). Apart from this strategy, the use of unfamiliar linguistic items in the experiment might bring more promising results in order to not misinterpret children's existing knowledge. Consistent with what was investigated in this study, we are encouraged to make use of other strategies that might build a cognitive learning scenario with the presence of a social robot. We believe that interactions with the robot can involve several modes (verbal, visual, tactile) and be integrated in relation to all perceptual modalities, together with events on the tablet, its stylus data, and children's feedback.

### 6.6. Handwriting Recognition

To measure the children's handwriting performances, we developed handwriting recognition for the Cyrillic alphabet. The accuracy rate on a validation set using state-of-the-art algorithms, i.e., 784-500-500-2000 reported in Hinton and Salakhutdinov (2006) and CNN similar to Le-Net-5 (LeCun et al., 1998) with custom parameters is 98% on the Cyrillic-MNIST data set. However, the recognition of children's handwriting data was only 38%. This is reflected in other works that use adult datasets with child data: state-of-the-art speech recognition technologies (Kennedy et al., 2017) did not perform well with child speech, while age and gender determination did not perform well on children's faces (Sandygulova et al., 2014). As noted by Asselborn et al. (2018), the quality of handwriting performance can only be evaluated when considering the age and gender of children. The collection of a dataset on children's Cyrillic handwriting will, subsequently, allow us to adequately evaluate the quality of Cyrillic handwriting in real-time.

## 7. Conclusion

In this paper, the CoWriting Kazakh system and its proposed learning scenario were discussed in relation to the script conversion task in Kazakh, from Cyrillic to Latin in three conditions (a robot, tablet, and a teacher). Contributing to the HRI field, the main findings that can be drawn from this interdisciplinary study are: (1) tablets only and tablets along with robots have the potential to provide more-or-less similar learning gains as a teacher in the script learning scenario with children, since the three conditions did not show significant differences, however (2) robots are advantageous based on the significant positive mood change and children's responses that they liked the robot significantly more and considered it as significantly more interesting than other learning aids in the present study and in conclusions reached by previous studies (Park and Howard, 2013; Westlund et al., 2015; Vogt et al., 2019), (3) an open question remains as to whether gender difference is significant in regard to learning outcomes. Our study could not reach definitive conclusions since there were several limitations such as single-session intervention, relatively small sample size and the lack of only robot condition. However, there is an overall lack of such studies in the field of HRI that compare effectiveness of robotic systems as opposed to other learning aids. In essence, social robots can significantly impact children's learning as they tend to cultivate a responsive and friendly interaction (Belpaeme et al., 2018a; Kanero et al., 2018). Considering all the above, our future studies should aim for longitudinal interaction and further investigate gender difference, differentiated learning, the refinement of learning scenarios related to word choice, and adaptations for remote and online learning. We hope this study will increase scholarly attention towards the use of robots for script learning and handwriting practice. In order to effectively teach the Latin-based Kazakh alphabet, our essential purpose is to develop an adaptive system relying on differentiated learning strategies relevant to various learning scenarios and individuals.

## Data Availability Statement

The raw data supporting the conclusions of this article will be made available by the authors, without undue reservation.

## Ethics Statement

The studies involving human participants were reviewed and approved by Nazarbayev University Institutional Research Ethics Committee. Written informed consent to participate in this study was provided by the participants' legal guardian/next of kin. Written informed consent was obtained from the individual and minor's legal guardian for the publication of any potentially identifiable images or data included in this article.

## Author Contributions

ZZ: investigation, data curation, visualization, and writing. AA: writing. MA, KK, and NB: investigation and data curation. BT and TA: software. WJ and PD: conceptualization, supervision, and funding acquisition. AC: supervision, funding acquisition, and writing. AS: conceptualization, supervision, funding acquisition, writing, investigation, and data curation. All authors contributed to the article and approved the submitted version.

## Conflict of Interest

The authors declare that the research was conducted in the absence of any commercial or financial relationships that could be construed as a potential conflict of interest.

## Footnotes

^1^https://github.com/chili-epfl/cowriter
